# Supplementary material for: Molecular Landscape of the Coagulome of Oral Squamous Cell Carcinoma
Source: Cancers (Basel). 2022 Jan 17;14(2):460. doi: 10.3390/cancers14020460 (PMC8773794; doi:10.3390/cancers14020460)

cancers

MDPI

Article

Molecular Landscape of the Coagulome of Oral Squamous Cell Carcinoma

Marine Lottin, Simon Soudet, Julie Fercot, Floriane Racine, Julien Demagny, Jérémie Bettoni, Denis Chatelain, Marie-Antoinette Sevestre, Youcef Mammeri, Michele Lamuraglia, Antoine Galmiche and Zuzana Saidak

Supplementary Materials

Table S1. Clinical characteristics of OSCC patients in TCGA ($n = 320$).

**Median age (years)**	**61**
Sex	Female n = 107; Male n = 213
Primary site	Alveolar ridge n = 18
	Buccal mucosa n = 23
	Floor of Mouth n = 63
	Hard palate n = 7
	Lip n = 3
	Oral cavity n = 73
	Oral tongue n = 133
pTNM	Stage I n = 21; Stage II n = 54; Stage III n = 57; Stage IV n = 165; NA n = 23
	T T1 n = 31; T2 n = 99; T3 n = 61; T4 n = 111; TX n = 9; NA n = 9
	N N0 n = 122; N1 n = 48; N2 n = 103; N3 n = 3; NX n = 34; NA n = 10
	M M0 n = 121; MX n = 34; NA n = 165
Alcohol history	NO n = 107; YES n = 206; NA n = 7
Smoking category	Non-smoker n = 89; Current smoker n = 97; former smoker n = 125; NA n = 9
ALI	NO n = 168; YES n = 71; NA n = 81
ECS	None n = 173; microscopic n = 47; gross n = 20; NA n = 80
PNI	NO n = 114; YES n = 138; NA n = 68
Surgical margin	Negative n = 234; Close n = 36; positive n = 37; NA n = 13

ALI: Angiolymphatic Invasion; ECS: Extracapsular Spread; PNI: Perineural invasion.

Table S2. Frequency of alterations/DNA seq in OSCC in TCGA (n = 315).

	# **mutations (%)**	# **amplifications (%)**	# **deep deletions (%)**
F3	2 (0.64)	1 (0.32)	0 (0)
PLAU	3 (0.96)	3 (0.95)	0 (0)
PLAT	4 (1.27)	7 (2.22)	3 (0.95)
PLAUR	0 (0)	1 (0.32)	0 (0)
F2R	2 (0.64)	0 (0)	1 (0.32)
SERPINE1	6 (1.91)	7 (2.22)	0 (0)

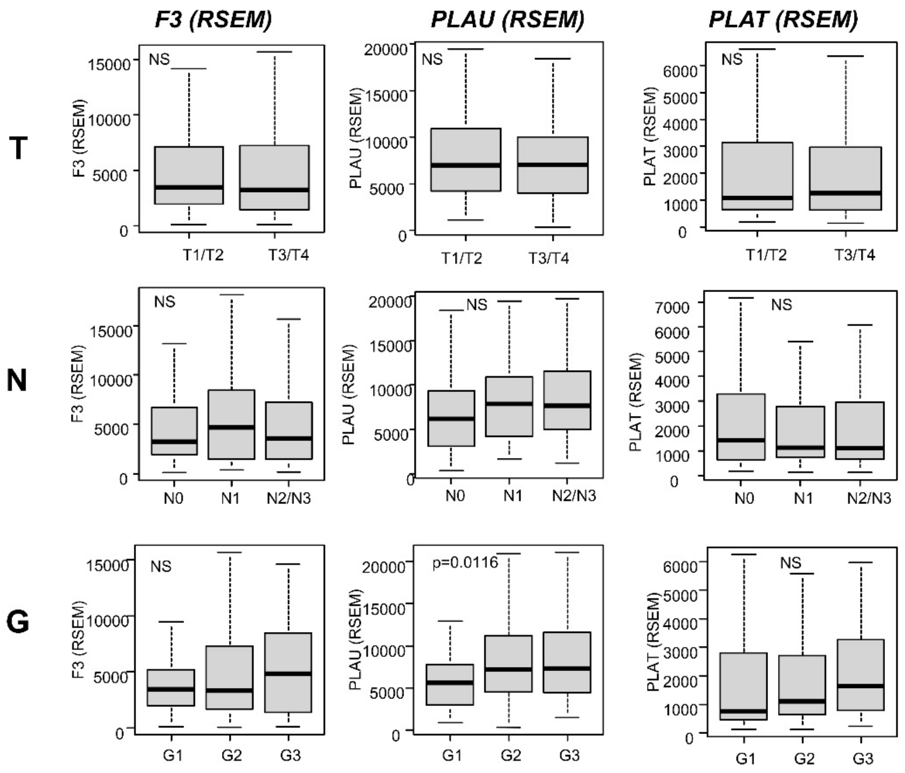

Figure S1. Expression of the coagulome and association with TNM/grade in OSCC. Clinical correlates of F3, PLAU and PLAT mRNA expression in OSCC (n = 315 with genomic and clinical data). Tumors were analysed by size T (T1/2 n = 128 vs. T3/4 n = 169), by nodal involvement N (N0 n = 120 vs. N1 n = 47 vs. N2/3 n = 103) and by grade (G1 n = 49 vs. G2 n = 196 vs. G3 n = 66). Wilcoxon or Kruskal Wallis tests were used as appropriate. $p < 0.05$ was set as the threshold for significance.

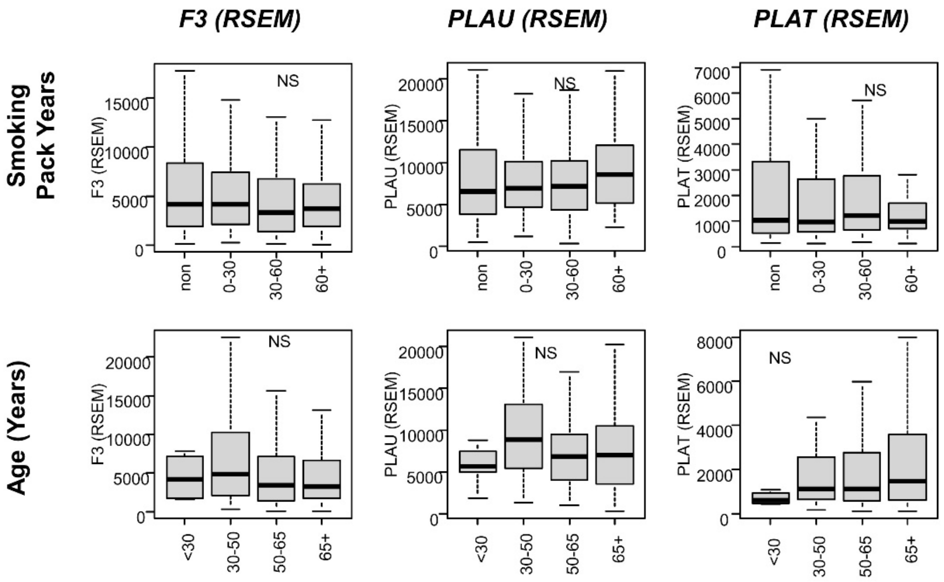

Figure S2. Expression of the coagulome and association with age and the smoking status in OSCC. Boxplots showing a lack of association between *F3, PLAU* and *PLAT* mRNA expression (RSEM) and the smoking history (pack years, upper panel) and patient age (years, lower panel) in OSCC patients from TCGA. A Kruskal Wallis test was performed to evaluate the differences between conditions. $p < 0.05$ was set as threshold for significance.

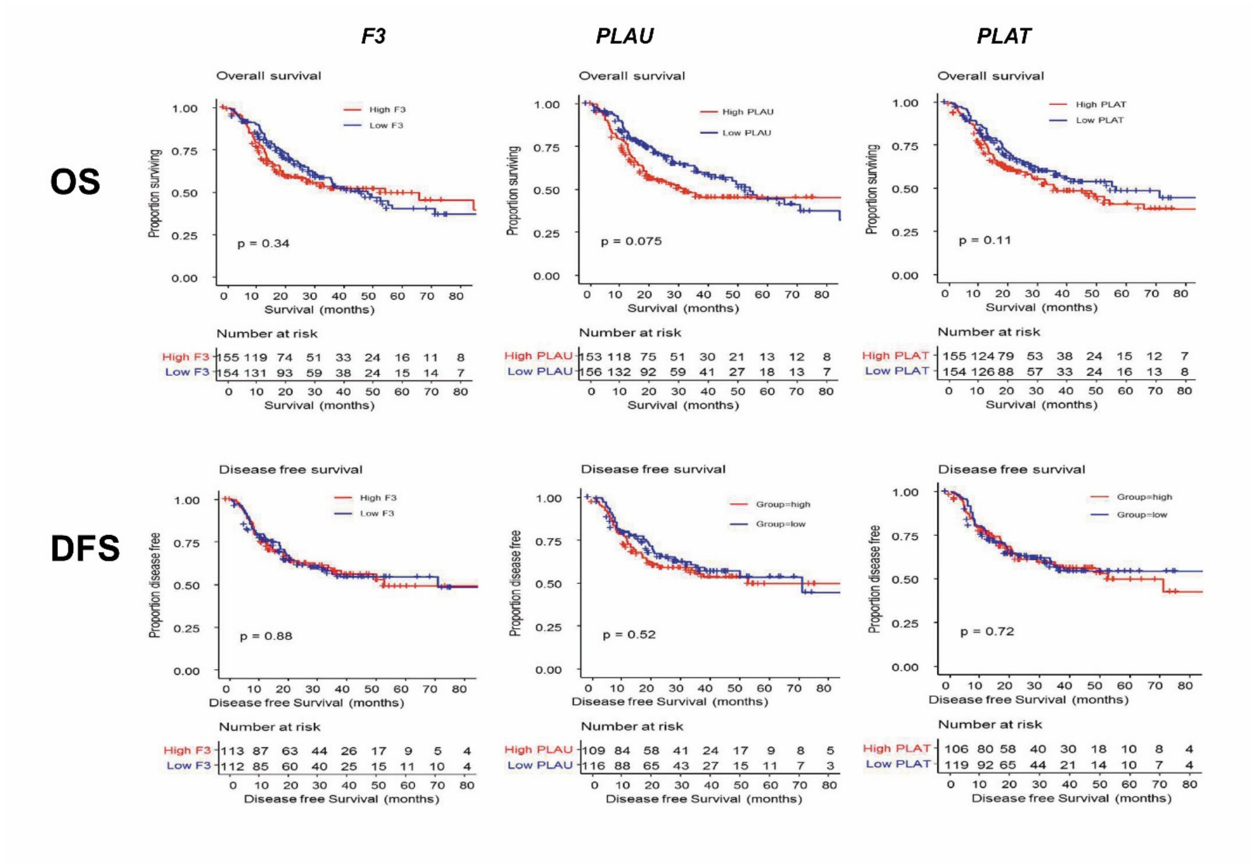

Figure S3. Link between *F3*, *PLAU* and *PLAT* gene expression and OSCC patient survival. Kaplan-Meier analysis comparing the OS and DFS in OSCC patients from TCGA stratified in each case into two groups according to *F3*, *PLAU* or *PLAT* by the median. High expressors are shown in red, low expressors in blue. A lack of association between *F3*, *PLAU* and *PLAT* gene expression and OSCC patient survival was demonstrated using the log-rank test, setting $p < 0.05$ as threshold for significance.

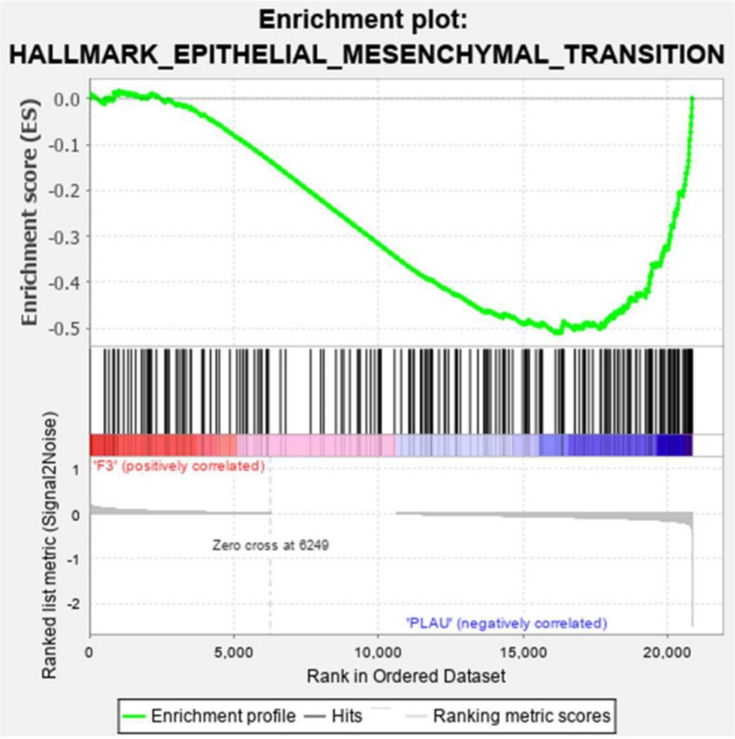

Figure S4. GSEA analysis of F3 expressing cancers cells vs. PLAU expressing cancer cells. GSEA analysis of a representative tumor from Puram et al. (GSE103322) comparing *F3* expressing cells (n = 246) vs. *PLAU* expressing cells (n = 105). GSEA analysis revealed the enrichment of the «Hallmark Epithelial Mesenchymal Transition» gene set (NES = 1.84, p = 0.0066 (FDR)).

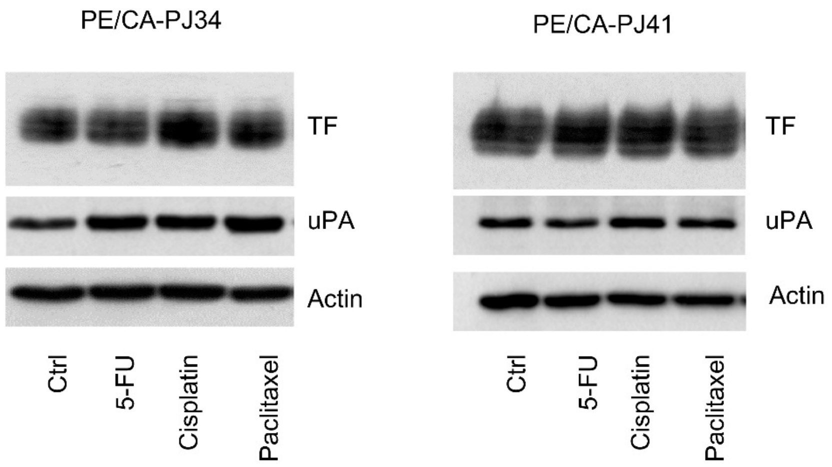

Figure S5. Immunoblot analysis of TF, uPA and PAI-1 in OSCC cells exposed to chemotherapeutic agents. The cell lines PE/CA-PJ34 and PE/CA-PJ41 were exposed to the chemotherapeutic agents 5-FU, cisplatin and paclitaxel applied for 48 h at a concentration of 10 µM, 10 µM and 0.1 µM, respectively (corresponding to the IC_{50} for each drug). Actin is given as control.

Original Western Blots

Figure 10A

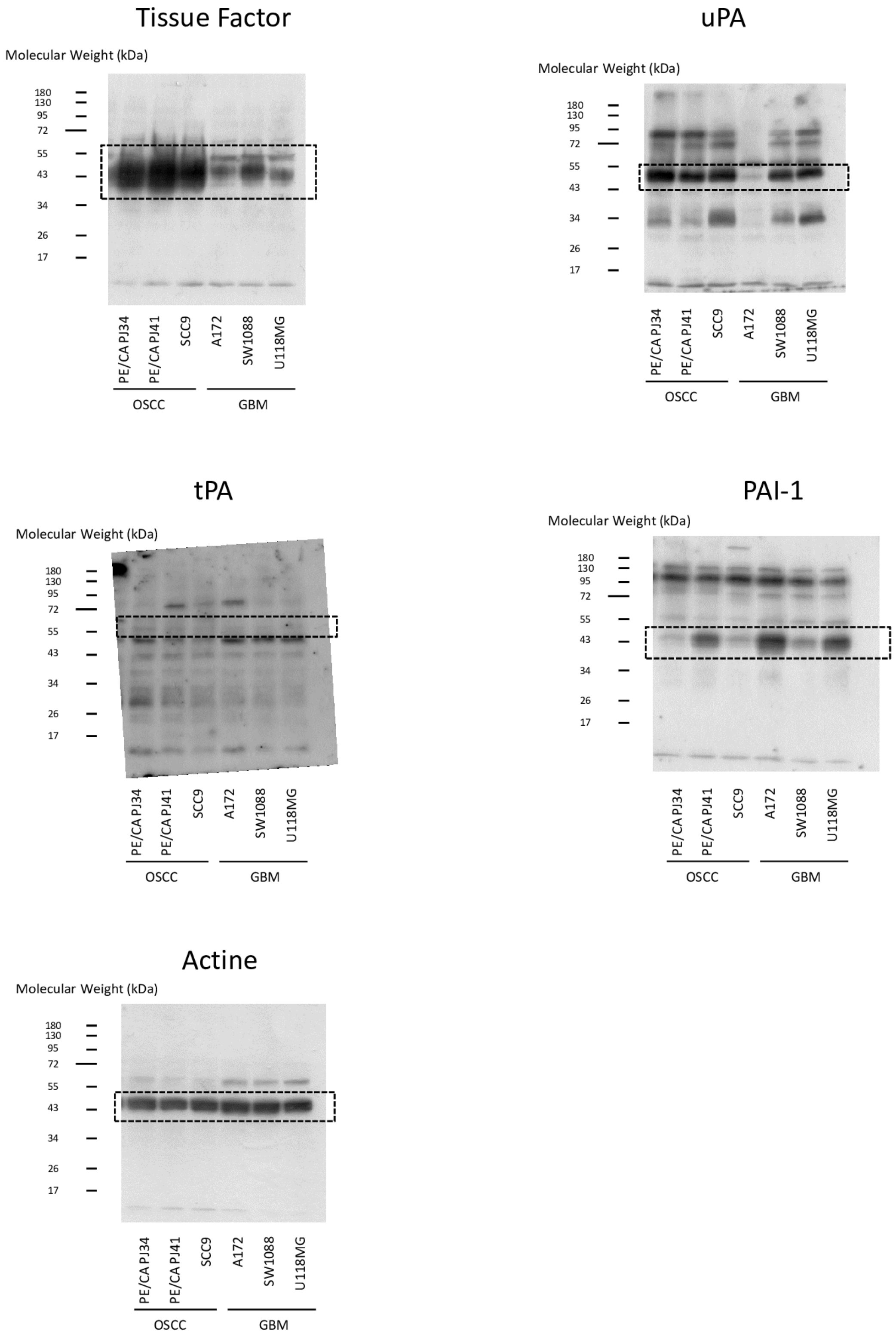

Figure 10C

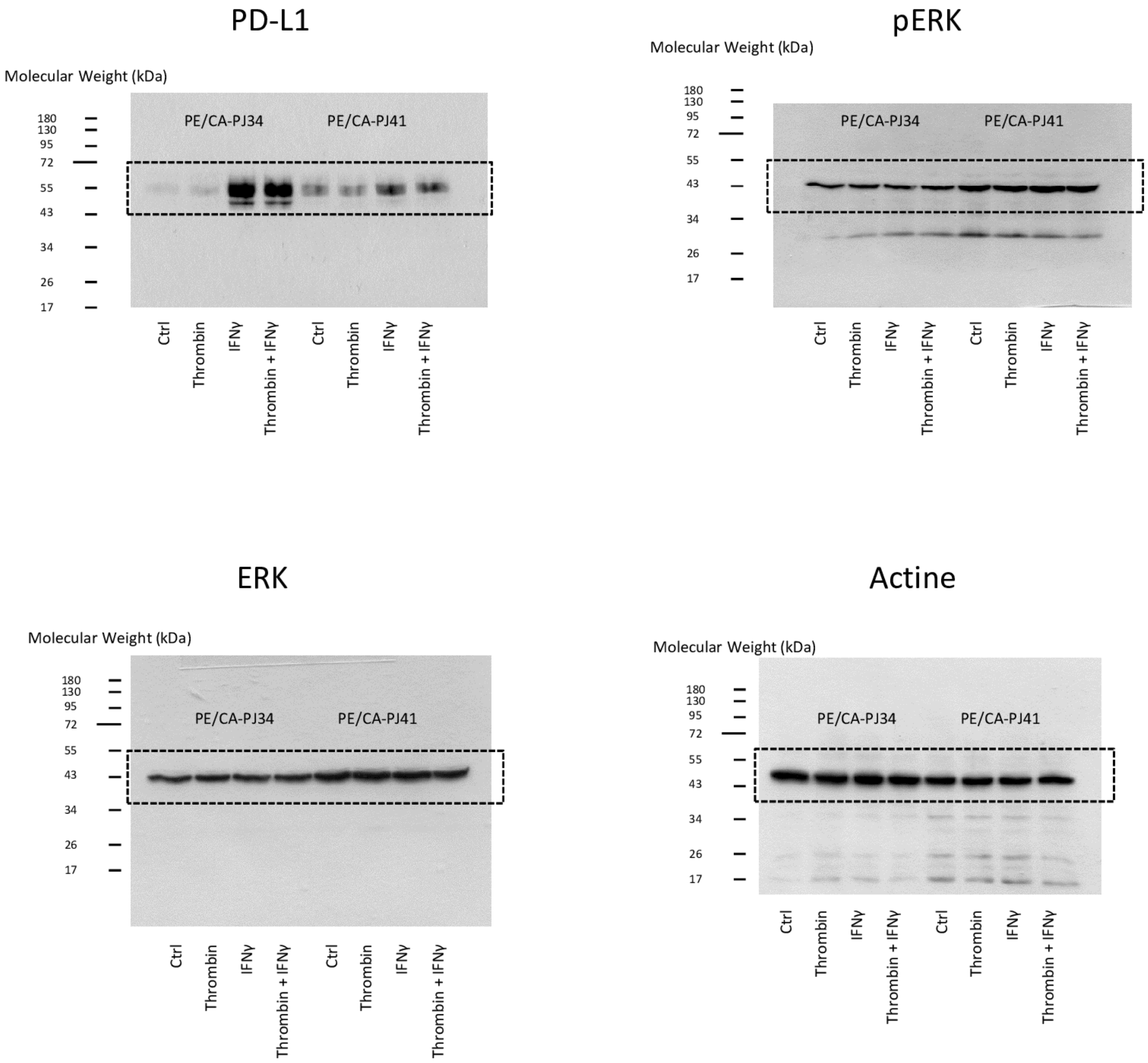
PD-L1
Molecular Weight (kDa)
180
130
95
72
55
43
34
26
17
PE/CA-PJ34
PE/CA-PJ41
Ctrl
Thrombin
IFNγ
Thrombin + IFNγ
Ctrl
Thrombin
IFNγ
Thrombin + IFNγ
pERK
Molecular Weight (kDa)
180
130
95
72
55
43
34
26
17
PE/CA-PJ34
PE/CA-PJ41
Ctrl
Thrombin
IFNγ
Thrombin + IFNγ
Ctrl
Thrombin
IFNγ
Thrombin + IFNγ
ERK
Molecular Weight (kDa)
180
130
95
72
55
43
34
26
17
PE/CA-PJ34
PE/CA-PJ41
Ctrl
Thrombin
IFNγ
Thrombin + IFNγ
Ctrl
Thrombin
IFNγ
Thrombin + IFNγ
Actine
Molecular Weight (kDa)
180
130
95
72
55
43
34
26
17
PE/CA-PJ34
PE/CA-PJ41
Ctrl
Thrombin
IFNγ
Thrombin + IFNγ
Ctrl
Thrombin
IFNγ
Thrombin + IFNγ

Suppl. Figure 5

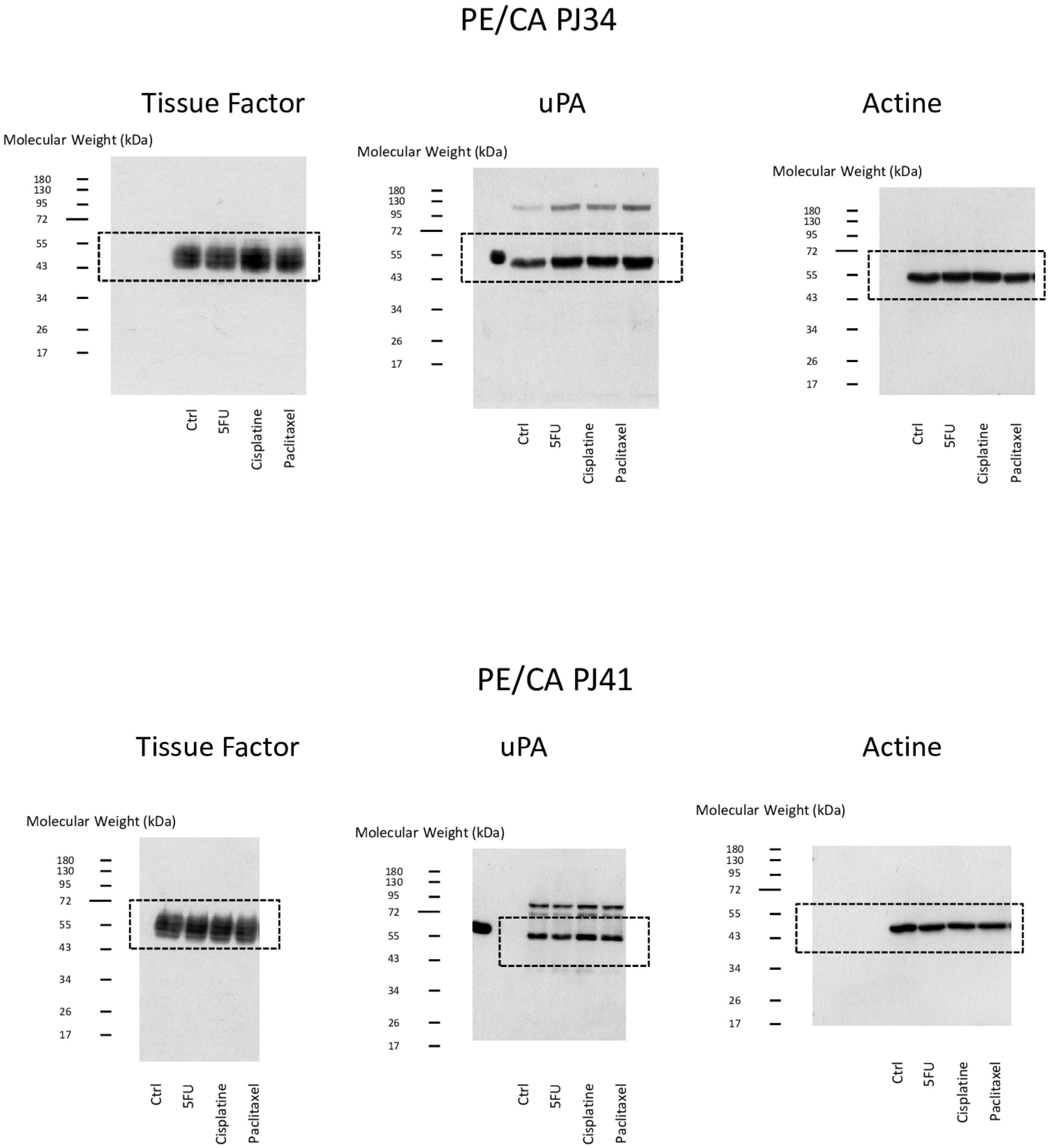

Supplement: Supplementary file 1 [file cancers-14-00460-s001.zip › cancers-1537175-supplementary.pdf]
